# Anthraquinone Derivative Rufigallol Protects Against Ethanol-Induced Gastric Damage via Modulation of PGE_2_, NO, and Inflammatory Pathways: In Vivo and In Vitro Study

**DOI:** 10.3390/antiox15070869

**Published:** 2026-07-12

**Authors:** Tariq G. Alsahli, Khushhaal, Sami I. Alzarea, Hesham A. M. Gomaa, Muhammad Afzal

**Affiliations:** 1 Department of Pharmacology, College of Pharmacy, Jouf University, Sakaka 72341, Aljouf, Saudi Arabia; tgalsahli@ju.edu.sa (T.G.A.); samisz@ju.edu.sa (S.I.A.);; 2 Glocal School of Life & Allied Health Sciences, Glocal University, Saharanpur 247121, Uttar Pradesh, India; 3 Department of Pharmaceutical Sciences, Pharmacy Program, Batterjee Medical College, Jeddah 21442, Makkah, Saudi Arabia

**Keywords:** anthraquinone, antioxidant, ethanol, gastric ulcer, inflammation, oxidative stress

## Abstract

Excessive ethanol consumption is a cause of gastric ulceration. This study aimed to identify the gastroprotective action of rufigallol, an anthraquinone, against ethanol-induced gastric ulcers and assess its antioxidant and anti-inflammatory properties. Healthy rats were grouped into five groups (*n* = 8); the control group received sterile saline orally; the ethanol group received ethanol (5 mL/kg) to generate gastric ulcers on the last day of the experiment; the pretreated rufigallol group was administered 10 or 20 mg/kg rufigallol orally for a week before gastric ulcer initiation; and the drug control group received omeprazole (20 mg/kg) for a week with ethanol treatment. The results showed that oral rugalallol significantly reduced gastric ulcers, as indicated by decreased gastric juice volume and increased preventive percentage, gastric pH value, and pepsin activity. Histopathology confirmed a reduction in the gastric ulcer index following rufigallol treatment. Rufigallol pre-treatment significantly increased antioxidant levels (CAT, SOD, and GSH) and decreased MDA levels compared to the ethanol group. Furthermore, rufigallol treatment decreased MPO, pro-inflammatory cytokines, and mediator levels. It also reduced COX-2, IFN-γ, and NLRP3 expression and restored NO and PGE2 levels. In vitro experiments using HT-29 and HaCaT cell lines confirmed that rufigallol reduced cytokine production and exhibited anti-inflammatory activity in response to lipopolysaccharide stimulation. These results suggested that rufigallol administration may provide gastroprotective benefits against ethanol-induced gastric ulcers, potentially by reducing oxidative stress and gastric inflammation.

## 1. Introduction

Gastric ulcer (GU), a type of peptic ulcer disease, is a prevalent global health condition characterized by erosion of the stomach lining due to an imbalance between protective and destructive factors [1]. Despite advances in treatment, peptic ulcer disease, which includes both gastric and duodenal ulcers, remains a significant cause of illness worldwide [2]. Major global determinants for the development of GUs contain persistent *Helicobacter pylori* infection and prevalent application of non-steroidal anti-inflammatory drugs, both of which disrupt mucosal integrity and promote ulcer formation [2,3]. Ethanol-induced gastric ulcers (EI-GUs) pose a significant global health challenge largely due to widespread alcohol consumption, a primary risk factor for GU development [4]. Epidemiological data show that countries where people drink more alcohol have more cases of ethanol-related peptic ulcer and other problems, like bleeding and damage to the stomach lining. The risk of alcohol-related GU is increased by other lifestyle factors, such as smoking, stress, and other physiological and mental factors, which contribute to the global disease burden [5]. EI-GUs are commonly used as experimental models to study gastric mucosal injury caused by excessive alcohol consumption. Ethanol acts as a major pathogenic factor by causing direct damage to the gastric mucosa, leading to mucosal erosion, hemorrhage, inflammation, oxidative stress, and apoptosis in gastric tissues [6,7]. Ethanol also impairs mucosal defense by decreasing mucus secretion, reducing mucosal blood flow, and suppressing protective heat shock protein-70 expression, further exacerbating tissue injury [8].

The pathophysiology involves overproduction of reactive oxygen species (ROS), which eventually generate oxidative stress, leading to increased lipid peroxidation and downregulation of antioxidant enzymes and protective structures, such as superoxide dismutase (SOD), catalase (CAT), and glutathione (GSH). Ethanol exposure leads to the production of pro-inflammatory cytokines, including tumor necrosis factor-α (TNF-α), interleukin-1β (IL-1β), and IL-6, and activates nuclear factor kappa B (NF-κB) signaling, collectively disrupting mucosal integrity and promoting ulcer formation. The disruption of mucus production and mucosal glycoproteins also contributes to ulcer formation [6,7]. The participation of interferon-γ (IFN-γ) in ethanol-induced gastric ulceration is also intrinsically linked to its role as a pro-inflammatory cytokine that exacerbates local inflammation and epithelial damage through NF-κB-dependent pathways [9]. Moreover, enzymes such as inducible nitric oxide (NO) synthase are upregulated, contributing to nitrosative stress, whereas endothelial NO synthase is often downregulated during ethanol-induced injury [4]. Myeloperoxidase (MPO) activity is a critical marker of neutrophil infiltration and the inflammation cascade in the pathogenesis of EI-GU [10]. Ethanol exposure of gastric tissue enhances the expression and activation of the NLRP3 inflammasome, resulting in mucosal injury via the production of pro-inflammatory cytokines, including IL-1β and TNF-α. The onset of ulcers is associated with attenuated expression of protective components, such as gastric mucus and glycoproteins, NO, and prostaglandin E_2_ (PGE_2_), which typically preserve mucosal blood flow and integrity. Ethanol significantly reduces these components, thereby impairing mucosal defense [11,12]. In GU, apoptotic pathways are also triggered, sustained by enlarged expression of the caspase-3 and pro-apoptotic proteins such as BAX, whereas anti-apoptotic markers such as Bcl-2 are downregulated [8].

Various natural compounds and pharmacological agents have demonstrated gastroprotective effects against ethanol-induced ulcers, primarily through antioxidant, anti-inflammatory, and anti-apoptotic mechanisms. For instance, casuarinin [6], raspberry ketone [13], tert-butylhydroquinone [5], *Salvadora persica* (Miswak) [4], and fermented lotus root [9] significantly reduce ulcer area by enhancing mucin secretion, oxidant/antioxidant balance (GSH, CAT), and PGE_2_ levels, activating the Nrf2/HO-1 antioxidant signaling pathway, and subduing inflammatory mediators (NF-κB), pro-inflammatory cytokines, and apoptotic markers. Rufigallol is a naturally occurring anthraquinone derivative known for its beneficial activities, such as antioxidant and anti-inflammatory. Although detailed studies specifically on the antioxidant and anti-inflammatory activities of rufigallol are limited, it belongs to a class of compounds, anthraquinones and naphthoquinones, which are well documented to exhibit such properties [14]. This mechanism is central to their therapeutic potential because oxidative stress is a key driver of inflammation and related long-term diseases. In relation to their anti-inflammatory effects, naphthoquinones, a chemical family closely related to anthraquinones, modulate inflammatory pathways by inhibiting pro-inflammatory signaling, including NF-κB, a master controller of inflammation, and by reducing the production of pro-inflammatory mediators, such as NO and ROS. Given their structural similarity, rufigallol may exert comparable anti-inflammatory properties by downregulating key inflammatory mediators and pathways, thereby attenuating inflammation at the cellular level [15,16]. The potential antioxidant activity of rufigallol supports its anti-inflammatory role, as oxidative stress and inflammation are interlinked processes. This dual action is common in natural polyphenols and quinones, making rufigallol a promising agent for managing oxidative stress-related inflammation [16,17].

Several studies have reported the antioxidant and anti-inflammatory properties of rufigallol, an anthraquinone. However, its potential protective effects against ethanol-induced gastric ulceration have not been systematically investigated. Specifically, information regarding its ability to modulate oxidative stress, inflammatory mediators, and gastric histopathological alterations in experimental gastric injury is limited, and supporting in vitro evidence for its anti-inflammatory activity is scarce. Thus, this study aimed to evaluate the gastroprotective effect of rufigallol in an ethanol-induced gastric ulcer model in rats and investigate its impact on oxidative stress biomarkers, inflammatory cytokines, and histopathological changes. Additionally, we assessed the anti-inflammatory activity of rufigallol in an LPS-stimulated cell model by measuring cell viability and IL-6 and TNF-α production. Integrating in vivo and in vitro approaches, this study provides new experimental evidence supporting the gastroprotective and anti-inflammatory potential of rufigallol in acute gastric injury.

## 2. Materials and Methods

### 2.1. Reagents and ELISA Kits

Rufigallol was procured from MSW Pharma (Maharashtra), India. For this research, kits like rat NO (Cat. No. MBS2540417; Detection range 0.97–700 µmol/L; Sensitivity typically up to 0.97 µmol/L), PGE_2_ (Cat. No. MBS262150; Detection range 15.6–1000 pg/mL; Sensitivity typically up to 5 pg/mL; intra-assay CV ≤ 8%; inter-assay CV ≤ 12%), COX-2 (Cat No. MBS266603; Detection range 0.156–10 ng/mL; Sensitivity typically up to 0.05 ng/mL; intra-assay CV ≤ 8%; inter-assay CV ≤ 12%), TNF-α (Cat No. MBS282960; Detection range 6.25–400 pg/mL; Sensitivity typically < 3.1 pg/mL; intra-assay CV ≤ 8%; inter-assay CV ≤ 12%), IL-1β (Cat. No. MBS265868; Detection range–15.6–1000 pg/mL; Sensitivity usually < 5 pg/mL; intra-assay CV ≤ 8%; inter-assay CV ≤12%), IL-6 (Cat. No. MBS269892; Detection range 15.6–1000 pg/mL; Sensitivity up to 5 pg/mL; intra-assay CV ≤ 8%; inter-assay CV ≤ 12%), IFN-γ (Cat No. MBS2019134; Detection range 15.6–1000 pg/mL; Sensitivity typically ≤ 6.1 pg/mL; intra-assay CV ≤ 10%; inter-assay CV ≤ 12%), NF-κB (Cat No. MBS287521; Detection range 0.31–20 ng/mL; Sensitivity typically ≤ 0.15 ng/mL; intra-assay CV ≤ 8%; inter-assay CV ≤ 12%) and NLRP3 (Cat. No. MBS2033695; Detection range 0.312–20 ng/mL; Sensitivity typically < 0.115 ng/mL; intra-assay CV ≤ 10%; inter-assay CV ≤ 12%), were purchased from MyBioSource, Inc., San Diego, CA, USA.

### 2.2. Animals

The Wistar rats (180 ± 20 g, 10–12 weeks old, *n* = 8 per group) were cared for, fed, housed, and enriched at the animal center and research laboratory of Batterjee Medical College in Jeddah, Saudi Arabia. The rodents were kept on a 12 h light/dark cycle, maintained at 22 ± 2 °C and 40–50% relative humidity. Rats received standard pellets and unlimited water. The study was conducted in accordance with the ARRIVE guidelines. All experimental procedures were conducted in accordance with the Institutional Research Board of Batterjee Medical College in Jeddah, under the designated approval number-RES-2026-0063 (06/01/26).

### 2.3. Acute Oral Toxicity

The acute oral toxicity (LD50) of rufigallol was evaluated in accordance with the OECD Test Guideline 423 [18]. Rufigallol (10 or 20 mg/kg) was administered orally at the highest dose, as previously described. At these doses, it was reported to be safe and non-toxic in rats, aligning with its established safety profile [19]. The pkCSM tool was used to evaluate the pharmacokinetic (PK) properties, absorption, distribution, metabolism, excretion, and toxicity (ADMET) of rufigallol [20].

### 2.4. Experimental

**Figure 1:**
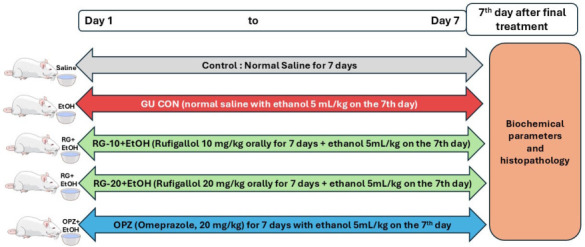
Experimental Design.

The rats (a total of 40) were randomly separated into 5 groups (*n* = 8): control group (CON; provide normal saline), gastric ulcer group (GU CON; normal saline with ethanol on the 7th day), treatment rufigallol-10 group (RG-10 + EtOH; Rufigallol 10 mg/kg orally for 7 days + ethanol), treatment rufigallol-20 group (RG-20 + EtOH; Rufigallol 20 mg/kg orally for 7 days + ethanol), and an omeprazole group that received omeprazole (OPZ; 20 mg/kg) for 7 days with ethanol treatment. A schematic scheme of the experimental strategy is shown in Figure 1. After 60 min, all rats except the control group received an oral dose of absolute alcohol (5 mL/kg) to induce gastric ulceration [21,22]. One hour later, the rats were anesthetized by the use of ketamine (87 mg/kg) and xylazine (12 mg/kg). Blood was then collected via intracardial perforation, and the rats were sacrificed following ethical guidelines. Macroscopic evaluation included determination of the ulcer index, gastric juice volume, gastric pH, and pepsin activity. Oxidative stress status was assessed by measuring malondialdehyde (MDA), superoxide dismutase (SOD), CAT, GSH, NO, and MPO activity in gastric tissue homogenates. Inflammatory responses were evaluated through quantification of TNF-α, IL-1β, IL-6, NF-κB, COX-2, IFN-γ, NLRP3, and prostaglandin E2 (PGE2) using ELISA-based methods. Histopathological examination of gastric tissues was performed using hematoxylin and eosin staining to assess mucosal injury, inflammatory cell infiltration, edema, necrosis, and tissue architecture. Additionally, in vitro studies were performed using HT-29 and HaCaT cell lines to assess cell viability (MTT assay) and cytokine production (TNF-α and IL-6) in response to lipopolysaccharide (LPS) stimulation. These experiments aimed to examine the anti-inflammatory effects of rufigallol in LPS-induced inflammatory conditions and to support the in vivo gastroprotection study. The chosen cell line is commonly used to analyze inflammatory responses and cytokine secretion following LPS stimulation, providing a consistent model for testing the anti-inflammatory activity of candidate compounds. Although gastric epithelial cell lines would better represent gastric mucosal injury, the current in vitro study focused on evaluating the general anti-inflammatory properties of rufigallol rather than directly modelling gastric epithelial damage.

### 2.5. Tissue Preparation

We homogenized stomach sections in 5 mL phosphate-buffered saline (PBS; pH 7.4). The resulting solution was then centrifuged at 10,000× *g* for 20 min at 4 °C. The clear upper debris-free solution was used for biochemical investigation. Additional portions were reserved at −80 °C for future examination of oxidative stress parameters. The collected supernatants were used for ELISA analysis. Total protein concentration was determined using the bicinchoninic acid (BCA) protein assay (MyBioSource, Inc., San Diego, CA, USA), and oxidative Stress, inflammatory responses, or cytokine concentrations were normalized to protein content. For further processing, specimens intended for histological analysis were submerged in 10% formalin.

### 2.6. Gastric Ulcer Index

The stomach sacs were opened along the greater curvature, washed and rinsed with cold normal saline, blotted dry using filter paper, and pinned flat on cardboard for gross examination of lesions. The ulcer index scores were allocated based on an arbitrary measure [23] with the comprehensive scoring methodology detailed in Table 1. The ulcer index was assessed by a blinded professional on gross stomach examination and the predefined ulcer severity score. The following formula measured the ulcer index:

$$UI\;=\frac{10}{X}\;$$

where UI = ulcer index; X = total area under ulceration.

The protective index (PI) was calculated using the following formula:

$$PI\;=\frac{ulcer\;model-ulcer\;treated}{ulcer\;model}\times 100\;$$

### 2.7. Gastric Juice pH Value and Volume

The contents extracted from the stomach were transferred into sterile centrifuge tubes and centrifuged at 2500× *g* for 600 s. The pH was subsequently measured with a digitally calibrated pH meter. The fluid content of the gastric mucosa was collected through centrifugation at 1000× *g* for 15 min. The separated upper liquid was then measured to determine the gastric volume [23].

### 2.8. Pepsin Activity

The stop-point bioassay, using the denatured hemoglobin hydrolysis technique, was performed to assess pepsin activity, and the methods were adapted from Alotaibi et al. [23].

### 2.9. Oxidative Stress (MDA, CAT, SOD, and GSH Level)

The excised stomach tissues from all experimental rodents were stored at −80 °C and subjected to biochemical estimation, as previously described. In this experiment, gastric tissue (~50 mg) was transformed into a powder and subsequently homogenized with PBS (500 μL). The prepared homogenate was used to assess oxidative stress-related indicators in the in vivo gastric tissue model, with parameters including MDA, SOD, CAT, GSH, and NO activities measured.

CAT activity in gastric tissue was determined by monitoring the rate of H_2_O_2_ decomposition at 240 nm. The enzymatic activity was subsequently calculated and expressed as units of CAT per milligram of tissue (U/mg tissue) [24].

To assess glutathione (GSH) activity in the serum supernatant, equal volumes of trichloroacetic acid were added, and the mixture was thoroughly mixed. Subsequently, 5,5′-dithiobis (2-nitrobenzoic acid) was added in equal portions and incubated for 30 min. The GSH activity was then quantified spectrophotometrically at 412 nm using a standard curve, with the results expressed as µmol GSH/mg protein [25].

The activity of superoxide dismutase (SOD) was evaluated utilizing a spectrophotometric method as defined by Nadeem et al. [26]. Briefly, the clear supernatant was mixed with a reagent solution and incubated for 30 min. Subsequently, nitroblue tetrazolium (NBT) was added, which forms a blue formazan complex and is measured by a spectrophotometer at 550 nm, and SOD activity was quantified and presented as units of SOD per mg of tissue (U/mg tissue).

The levels of malondialdehyde (MDA) in the collected supernatant were assessed through the thiobarbituric acid reactive substances (TBARS) method. The MDA concentration was determined using an extinction coefficient of 156,000/M/cm as described by Esterbauer and Cheeseman [27].

### 2.10. Quantification of Tissue Nitric Oxide (NO) and MPO Activity

The Griess reaction kit was utilized to quantify nitric oxide (NO) levels in gastric tissue homogenates. Colorimetric analysis was performed to quantify nitrite concentration, a stable product of NO. The absorbance of nitrite was measured at 540 nm and expressed as mM/g of tissue sample.

To quantify MPO activity, gastrointestinal lysate prepared in PBS (50 mM) was mixed with hexadecyltrimethylammonium bromide. The pH of this reaction mixture was maintained at 6. The resulting mixture was sonicated to avoid freezing. Afterwards, the mixture was centrifuged for 15 min at 4 °C at 12,000× *g*, and the clear supernatant was collected. The defined portion of clear supernatant was mixed with 0.005% hydrogen peroxide and o-dianisidine dihydrochloride to assess MPO activity [23].

### 2.11. Quantification of Inflammatory Markers (IL-6, IL-1β, TNF-α and NF-κB)

Sandwich ELISA kits with antibody capture were used to measure inflammatory markers, including IL-6, IL-1β, TNF-α, and NF-κB levels. The levels of inflammatory mediators were determined following the manufacturer’s instructions and are reported in pg/mL for IL-6, IL-1β, TNF-α, and NF-κB in the samples. The ELISA Kit, based on sandwich ELISA with antibody capture, was used to quantify inflammatory markers, including IL-6, IL-1β, TNF-α, and NF-κB levels. The kit manufacturer provided a standardized method that was strictly followed to perform the assay, and results were expressed in pg/mL for all the above markers.

### 2.12. Quantification of Prostaglandin E_2_ (PGE_2_) and NLRP3 Levels

The concentrations of PGE_2_ and NLRP3 in the stomach homogenate were measured using a standard commercial ELISA kit from MyBioSource, Inc., San Diego, CA, USA.

### 2.13. Assessment of Inflammatory Mediators

To measure the concentrations of IFN-γ and COX-2, both inflammatory mediators, ELISA kits using an antibody-capture-based sandwich technique were employed. Following the manufacturer’s guidelines, this method quantified the inflammatory mediators, with the results expressed in pg/mL.

### 2.14. Histopathological Examination Was Performed to Assess Mucosal Injury and Protection

A small sample of stomach tissue was taken from each rat and preserved in 4% paraformaldehyde for 24 h. It was then embedded in paraffin, sectioned, dewaxed, and rehydrated. The tissue was sliced into 5 μm thick sections and stained with hematoxylin and eosin. The samples were dehydrated, embedded, and processed as described by Mahdi et al. [28]. A blinded microscopic examination (Olympus Opto Systems India Pvt. Ltd., Noida, India) was conducted to assess and measure the severity of ulcer lesions and the extent of architectural restoration in each treatment group. Histopathological evaluation was performed by a single experienced observer, blinded to the treatment groups, to minimize observer bias. Since the analysis was conducted by one blinded evaluator, assessment of inter-observer agreement was not applicable.

### 2.15. Cell Culture

HT-29 human colon cancer epithelial cells derived from colorectal adenocarcinoma were used to assess the effect of rufigallol on cytokine production. HT-29 cells were recovered from liquid N_2_, defrosted at 37 °C using a water bath, and placed into a centrifuge tube with Roswell Park Memorial Institute (RPMI) 1640 medium supplemented with 10% fetal bovine serum. The cell mixture was centrifuged at 200× *g* for 10 min to isolate the cells. The separated cell pellet was then resuspended in complete medium and kept at 37 °C and 5% CO_2_. For the assessment of the effect of LPS (10 µg/mL) or rufigallol (at five different concentrations), the assay was applied. The five concentrations of rufigallol were selected to establish a clear dose–response relationship and identify the most effective, non-cytotoxic range in cell-based experiments.

### 2.16. MTT Assay

To evaluate the effects of rufigallol, 5000 cells in the logarithmic phase were seeded into each well of a 96-well plate. After 12 h, the control group received 1640 culture medium with 1% serum without rufigallol, while the test group received 1640 culture medium with 1% serum containing rufigallol at different concentrations. Following treatment, 100 µL of fresh medium containing 0.5 g/L MTT was added to each well, and the wells were incubated for 4 h at 37 °C under 5% CO_2_. The viable cells converted MTT into purple formazan crystals, which were dissolved in 0.1% dimethyl sulfoxide (DMSO) and quantified by measuring the OD at 490 nm with a microplate reader. Cell viability was calculated as:

$$Cell\;viability\;(\%)=\left(\frac{ODtreated}{ODcontrol}\right)\times 100\;$$

### 2.17. Quantification of Cytokines

To assess cytokine production in HT-29 cells, cells were exposed to rufigallol at five concentrations for 24 h. The five concentrations of rufigallol were selected to establish a clear dose–response relationship and identify the most effective, non-cytotoxic range in cell-based experiments. After 24 h of incubation, the cells were digested, and the cultures were centrifuged at 800× *g* for 5 min, and the clear supernatant was isolated. In the animal study component, colon tissue samples measuring 2–3 mm were collected and macerated in PBS. To ensure a clear sample for analysis, the homogenized solution was centrifuged at 12,000× *g* for 15 min. The levels of TNF-α and IL-6 in these supernatants were then quantified according to the kit manufacturer’s instructions.

### 2.18. Inhibition Assay

Immortalized human keratinocytes (HaCaT cells) were used to perform an inhibition assay to evaluate the anti-inflammatory activity of rufigallol under LPS stimulation. The five concentrations of rufigallol (20, 40, 60, 80, and 100 µg/mL) were selected to establish a dose–response relationship and identify the effective concentration range exhibiting anti-inflammatory activity without inducing cytotoxicity. This concentration range enabled evaluation of concentration-dependent effects on cell viability and cytokine production following LPS stimulation. HaCaT cells were cultured in Dulbecco’s Modified Eagle Medium (DMEM) complemented with 10% fetal bovine serum and 1% antibiotic–antimycotic solution. The cultures were maintained at 37 °C in 5% CO_2_. HaCaT cells were incubated in 24-well tissue culture plates at a concentration of 1 × 10^4^ cells/mL and permitted to adhere to the wall overnight. Prior to stimulation, the cells were pretreated with various concentrations of rufigallol for 2 h. The addition of LPS subsequently induced an inflammatory response. The cells were kept for 12 h in 37 °C in 5% CO_2_ atmosphere. In the control well, 0.2% DMSO in PBS was added along with cells and LPS, while untreated cells served as a normal control. The above experiments were done in triplicate. After 12 h, the supernatants from the culture well were isolated and centrifuged to remove cellular debris. TNF-α levels in the supernatants were determined using a commercially available ELISA immunoassay kit. Using a microplate reader, absorbance was measured at 450 nm, and the inhibition percentage of TNF-α and IL-6 production in the test samples was calculated relative to the LPS-treated control group.

### 2.19. Statistical Analysis

Statistical analyses were performed using GraphPad Prism (Version 8.0.2). Quantitative data are presented as mean ± SEM, and the ulcer index is presented as median with interquartile range. The Shapiro–Wilk test was used to assess normality. For normally distributed data, one-way ANOVA followed by Tukey’s post hoc test was used to determine variances between groups. Non-parametric data, specifically the ulcer index, were evaluated using the Kruskal–Wallis test. Results with a *p*-value < 0.05 were considered significant.

## 3. Results

### 3.1. Acute Toxicity Assessment

The selection of the 10 and 20 mg/kg doses for this study was based on the results of the 14-day toxicity study. Animals were observed for clinical signs of toxicity, behavioral changes, and mortality during the observation period in accordance with the acute oral toxicity protocol. No treatment-related abnormalities in general appearance, behavior, or survival were observed, indicating that rufigallol was well tolerated at the dose tested. Based on these findings and the available literature, doses of 10 and 20 mg/kg were selected as safe pharmacologically active doses to evaluate the gastroprotective effects of rufigallol while maintaining an adequate safety margin. The ADMET properties of rufigallol and its predicted _LD50_ for acute toxicity were 2.498 mol/kg.

### 3.2. Determination of Gastric Ulcer Index

**Figure 2:**
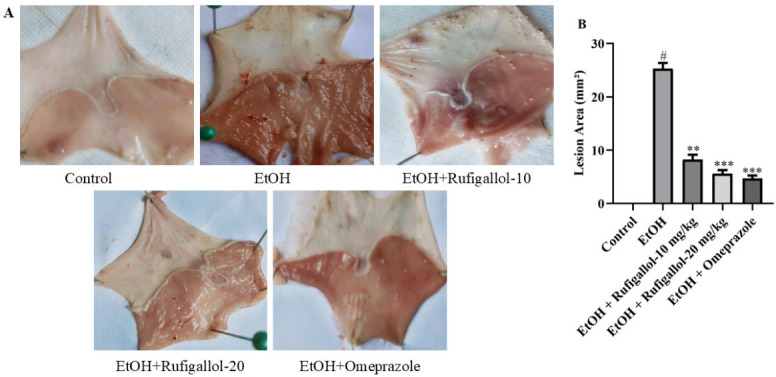
(**A**,**B**). (**A**) Representative high-resolution photographs showing the gastric mucosa of the experimental groups. Severe hemorrhagic lesions and mucosal damage were observed in the ethanol-treated group, whereas pretreatment with rufigallol (10 and 20 mg/kg) or omeprazole markedly reduced gastric injury. (**B**) Gastric ulcer area. Data are presented as mean ± S.E.M. (*n* = 8). Treatment groups: Control—Control untreated, EtOH: Gastric ulcer induced group only ethanol treated, EtOH + Rufigallol-10 mg/kg—Ethanol with co-treatment of 10 mg/kg rufigallol, EtOH + Rufigallol-20 mg/kg—Ethanol with co-treatment of 20 mg/kg rufigallol, and EtOH + Omeprazole—Ethanol with omeprazole (20 mg/kg) treatment. A one-way ANOVA followed by Tukey’s post hoc test, # *p* < 0.001 EtOH vs. control; ** *p* < 0.01, *** *p* < 0.001 rufigallol/omeprazole treated group vs. EtOH.

Figure 2A shows images of stomachs. According to Figure 2B, the ulcer area was 25.26 ± 1.09 mm^2^ in the ethanol-induced model. Treatment with rufigallol (10 and 20 mg/kg) efficiently reduced the abnormal varieties mentioned.

**Figure 3:**
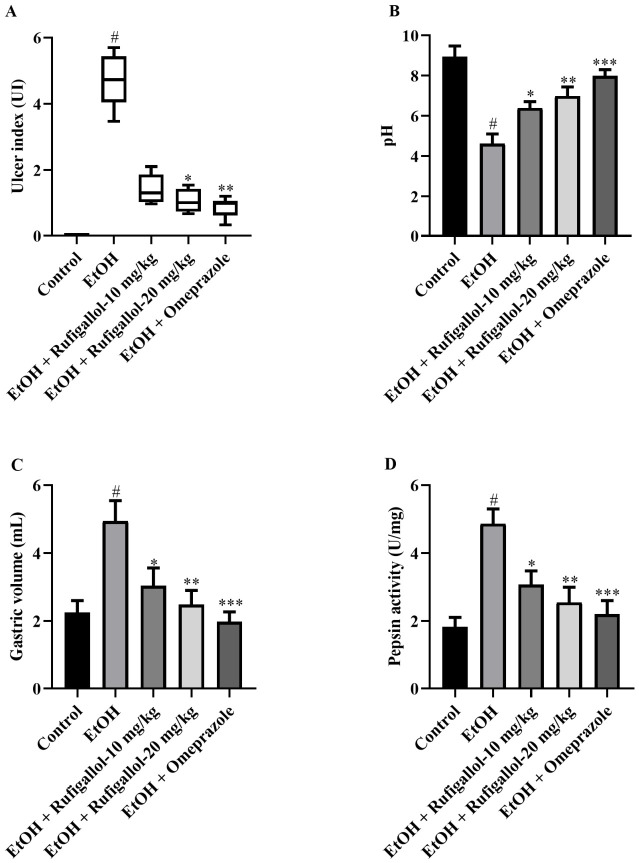
(**A**–**D**). Effects of rufigallol treatment on (**A**) Ulcer Index, (**B**) gastric pH, (**C**) gastric volume, and (**D**) Pepsin activity. Data are presented as mean ± S.E.M. (*n* = 8). Treatment groups: Control—Control untreated, EtOH: Gastric ulcer induced group only ethanol treated, EtOH + Rufigallol-10 mg/kg—Ethanol with co-treatment of 10 mg/kg rufigallol, EtOH + Rufigallol-20 mg/kg—Ethanol with co-treatment of 20 mg/kg rufigallol, and EtOH + Omeprazole—Ethanol with omeprazole (20 mg/kg) treatment. The ulcer index was evaluated using the Kruskal–Wallis test, and other parameters were analyzed using one-way ANOVA followed by Tukey’s post hoc test, # *p* < 0.001 EtOH vs. control; * *p* < 0.05, ** *p* < 0.01, *** *p* < 0.001 rufigallol/omeprazole treated group vs. EtOH.

In this study, the control group rats (ethanol) exhibited a higher UI than the untreated control group rats (*p* < 0.001). Rufigallol treatment at a low dose, that is, 10 mg/kg (*p*< 0.05), led to a noteworthy reduction in the ulceration index compared with ethanol-control rats. The rats that received a higher dose of 20 mg/kg (*p* < 0.01) showed a greater reduction in the ulcer index than those receiving the lower dose, indicating a dose-dependent effect (Figure 3A). Treatment with rufigallol at 10 and 20 mg/kg significantly reduced gastric lesions, producing protection indices of 69.98% and 77.58%, respectively. Omeprazole exhibited the highest gastroprotective effect with a protection index of 81.11% (Table 2).

### 3.3. Estimation of pH

Figure 3B represents the outcome of rufigallol action on pH in EI-GU in rats. In this study, the ethanol control group exhibited a notably lower pH than the normal control groups (*p* < 0.001) at the end of the experiment, that is, on the 8th day. The rufigallol-treated group that received a high dose (20 mg/kg) (*p* < 0.001) and a low dose (10 mg/kg) (*p* < 0.01) showed a notable improvement in bringing the alkaline pH parallel to that in the control group. Likewise, the rat groups that received omeprazole before ethanol induction exhibited a notable recovery to an alkaline pH compared with the ethanol control groups (*p* < 0.001).

### 3.4. Estimation of Total Acidity and Pepsin Activity

The ethanol-treated rat group exhibited a notable increase in total acid and pepsin activities compared with the control group (*p* < 0.001). Results discovered that the rufigallol-treated group (10 mg/kg) showed a slight decline in the total gastric volume among the treated group, the total acid (*p* < 0.05), and pepsin levels (*p* < 0.05) related to the ethanol-treated group of rats (Figure 3C,D). In contrast, the group treated with a higher dose (20 mg/kg) showed a significantly higher drop in total acid and pepsin levels (*p* < 0.01) compared to the low-dose-treated rats. Moreover, rats treated with omeprazole, the standard drug, during ethanol induction showed a significant increase in total acidity and pepsin levels compared with the ethanol-control group (*p* < 0.001).

### 3.5. Estimation of Oxidative Stress Markers

**Figure 4:**
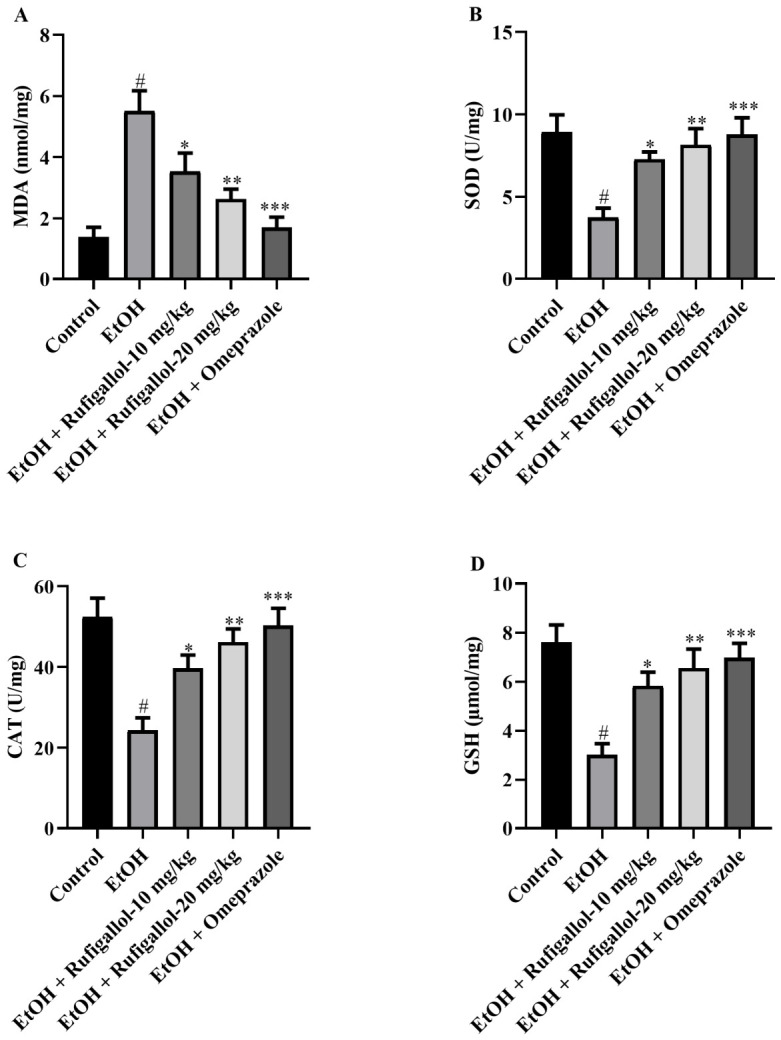
(**A**–**D**). Effect of rufigallol pre-treatment on oxidative stress markers. (**A**) MDA, (**B**) SOD, (**C**) CAT, and (**D**) GSH. Data are presented as mean ± S.E.M. (*n* = 8). Treatment groups: Control—Control untreated, EtOH: Gastric ulcer induced group only ethanol treated, EtOH + Rufigallol-10 mg/kg—Ethanol with co-treatment of 10 mg/kg rufigallol, EtOH + Rufigallol-20 mg/kg—Ethanol with co-treatment of 20 mg/kg rufigallol, and EtOH + Omeprazole—Ethanol with omeprazole (20 mg/kg) treatment. A one-way ANOVA followed by Tukey’s post hoc test, # *p* < 0.001 EtOH vs. control; * *p* < 0.05, ** *p* < 0.01, *** *p* < 0.001 rufigallol/omeprazole treated group vs. EtOH.

Treatment with rufigallol on EI-GU in rats affected MDA, SOD, CAT, and GSH levels in rats, as shown in Figure 4A–D. The ethanol-treated group of rats showed a significant increase in MDA levels (*p* < 0.001), while SOD, CAT, and GSH production showed a significant reduction (*p* < 0.001) compared with control rats. Treatment with both high and low doses of rufigallol (10 and 20 mg/kg) recovered the increased oxidative stress conditions by increasing in the SOD (*p* < 0.05 and *p* < 0.01), CAT (*p* < 0.05 and *p* < 0.01), and GSH (*p* < 0.05 and *p* < 0.01) levels in the treatment group and a reduction in MDA levels (*p* < 0.05 and *p* < 0.01) with respect to the ethanol-treated rats. The group of rats that received omeprazole showed noteworthy increases in SOD, GSH, and CAT levels (*p* < 0.001) and a decline in serum MDA levels compared with the ethanol-control group (*p* < 0.001). High recovery was observed under oxidative stress in the high-dose-treated group of rats compared with the low-dose-treated group, indicating dose-dependent recovery.

### 3.6. Assessment of NO and MPO Activity

**Figure 5:**
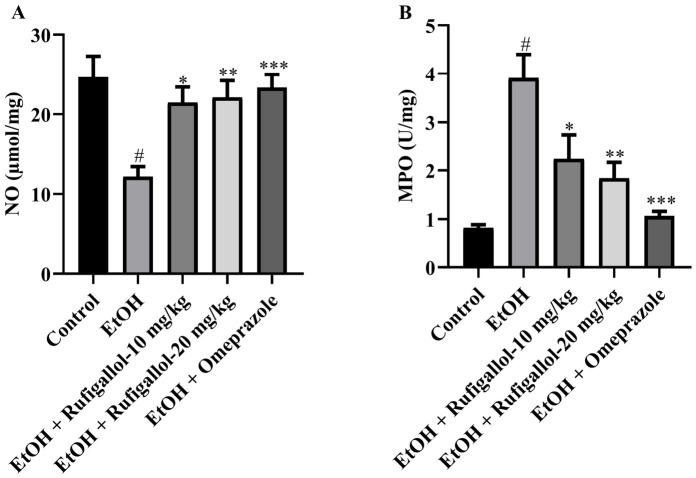
(**A**,**B**). Effect of rufigallol pre-treatment on oxidative stress markers. (**A**) NO, and (**B**) MPO activity. Data are presented as mean ± S.E.M. (*n* = 8). Treatment groups: Control—Control untreated, EtOH: Gastric ulcer induced group only ethanol treated, EtOH + Rufigallol-10 mg/kg—Ethanol with co-treatment of 10 mg/kg rufigallol, EtOH + Rufigallol-20 mg/kg—Ethanol with co-treatment of 20 mg/kg rufigallol, and EtOH + Omeprazole—Ethanol with omeprazole (20 mg/kg) treatment. A one-way ANOVA followed by Tukey’s post hoc test, # *p* < 0.001 EtOH vs. control; * *p* < 0.05, ** *p* < 0.01, *** *p* < 0.001 rufigallol/omeprazole treated group vs. EtOH.

Figure 5A,B shows the treatment effect of rufigallol on NO levels and MPA activity in EI-GU in rats. In this study, ethanol-induced control rats (*p* < 0.001) showed a notable reduction in NO levels, whereas MPO activity was markedly higher than in the control group (*p* < 0.001). Treatment with omeprazole and rufigallol at 10 mg/kg (*p* < 0.05) and 20 mg/kg (*p* < 0.001) remarkably restored NO levels, while MPO levels were lower than in the ethanol-treated group. Standard omeprazole treatment remarkably restores NO (*p* < 0.001) and MPO (*p* < 0.001) to levels similar to those of the control group.

### 3.7. Estimation of Pro-Inflammatory Markers and Mediators

**Figure 6:**
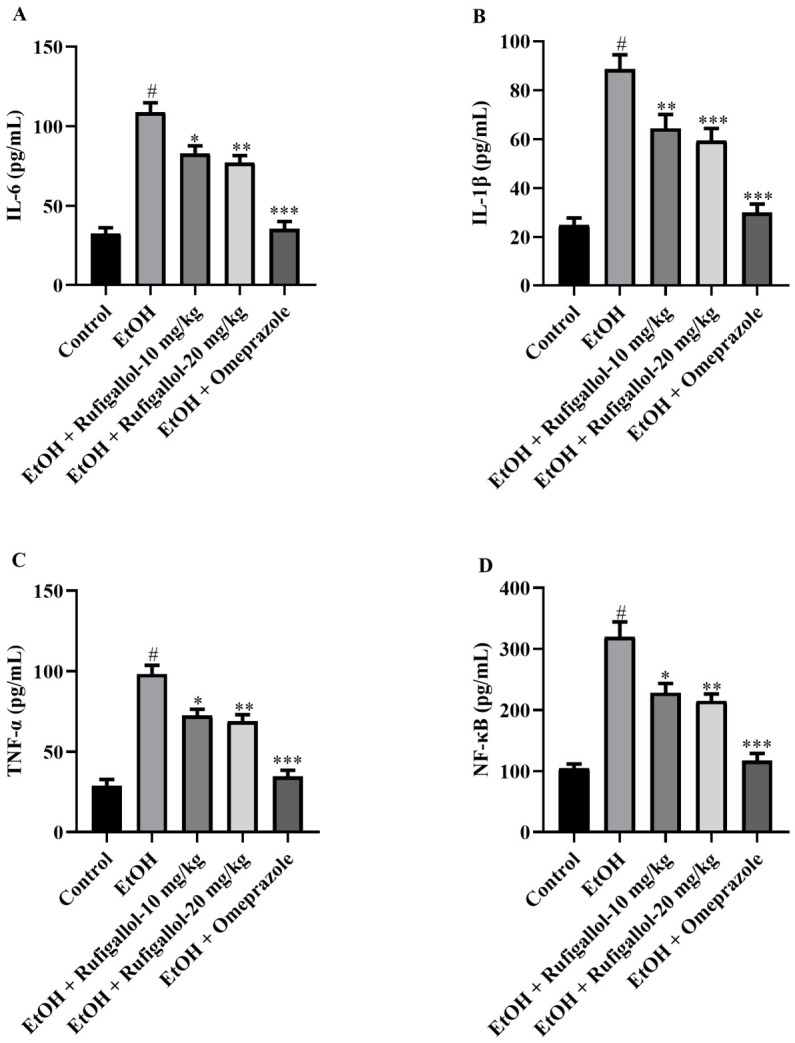
(**A**–**D**). Effect of rufigallol treatment on pro-inflammatory markers and mediator levels. (**A**) IL-6, (**B**) IL-1β, (**C**) TNF-α, and (**D**) NF-κB. IL-6—interleukin-6; IL-1β—interleukin-1β; TNF-α—Tumor necrosis factor-α; NF-κβ—Nuclear factor-κB. Data are presented as mean ± S.E.M. (*n* = 8). Treatment groups: Control—Control untreated, EtOH: Gastric ulcer induced group only ethanol treated, EtOH + Rufigallol-10 mg/kg—Ethanol with co-treatment of 10 mg/kg rufigallol, EtOH + Rufigallol-20 mg/kg—Ethanol with co-treatment of 20 mg/kg rufigallol, and EtOH + Omeprazole—Ethanol with omeprazole (20 mg/kg) treatment. A one-way ANOVA followed by Tukey’s post hoc test, # *p* < 0.001 EtOH vs. control; * *p* < 0.05, ** *p* < 0.01, *** *p* < 0.001 rufigallol/omeprazole treated group vs. EtOH.

Figure 6A–D represents the outcomes of rufigallol treatment in EI-GU in rats. The rats treated with ethanol showed notably higher levels of IL-6, IL-1β, TNF-α, and NF-κB (*p* < 0.001) than the untreated control group. Moreover, rufigallol treatment at low (10 mg/kg) and high (20 mg/kg) doses (*p* < 0.01) markedly downregulated IL-1β, IL-6, TNF-α, and NF-κB levels. The group of rats that received omeprazole (20 mg/kg; *p* < 0.001) during ethanol induction showed a marked decrease in these pro-inflammatory cytokines and markers. The decline in the levels of these pro-inflammatory cytokines and markers was greater in the group of rats treated with high doses (i.e., 20 mg/kg) than in the group treated with low doses (i.e., 10 mg/kg), indicating a dose-dependent treatment effect. The omeprazole treatment nearly restored all marker levels to control-group levels, as quantified.

### 3.8. Estimation of COX-2, PGE_2_, NLRP3 and IFN-γ Level

**Figure 7:**
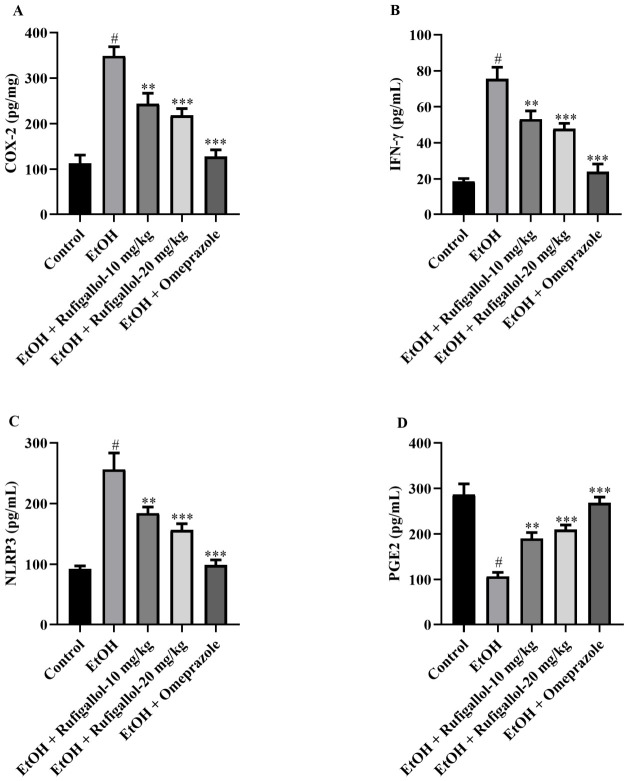
(**A**–**D**). Effect of rufigallol treatment on (**A**) COX-2, (**B**) IFN-γ, and (**C**) NLRP3 and (**D**) PGE_2_ levels. COX-2—Cyclooxygenase 2; IFN-γ –Interferon–γ; PGE_2_—prostaglandin E_2._ Data are presented as mean ± S.E.M. (*n* = 8). Treatment groups: Control—Control untreated, EtOH: Gastric ulcer induced group only ethanol treated, EtOH + Rufigallol-10 mg/kg—Ethanol with co-treatment of 10 mg/kg rufigallol, EtOH + Rufigallol-20 mg/kg—Ethanol with co-treatment of 20 mg/kg rufigallol, and EtOH + Omeprazole—Ethanol with omeprazole (20 mg/kg) treatment. A one-way ANOVA followed by Tukey’s post hoc test, # *p* < 0.001 EtOH vs. control; ** *p* < 0.01, *** *p* < 0.001 rufigallol/omeprazole treated group vs. EtOH.

The ethanol-induced group of rats showed significant increases in COX-2, NLRP3, and IFN-γ levels (*p* < 0.001) and a significant decrease in PGE2 levels (*p* < 0.001) compared with the control rats (Figure 7A–D). Treatment with rufigallol (at both doses, i.e., 10 and 20 mg/kg) and omeprazole showed a notable decline in COX-2, NLRP3, and IFN-γ levels, and a recovery in PGE2 levels, compared with the ethanol-induced ulcer group of rats. The decrease in these inflammatory markers was greater in rats administered high doses (i.e., 20 mg/kg; *p* < 0.001) than in those treated with the low dose (i.e., 10 mg/kg; *p* < 0.01), demonstrating a dose-dependent treatment effect. In the omeprazole-administered group of rats (*p* < 0.001), the levels of all these markers were almost equal to those in the control group of rats.

### 3.9. Effect of the Treatment of Rufigallol on the GU and Its Histology

**Figure 8:**
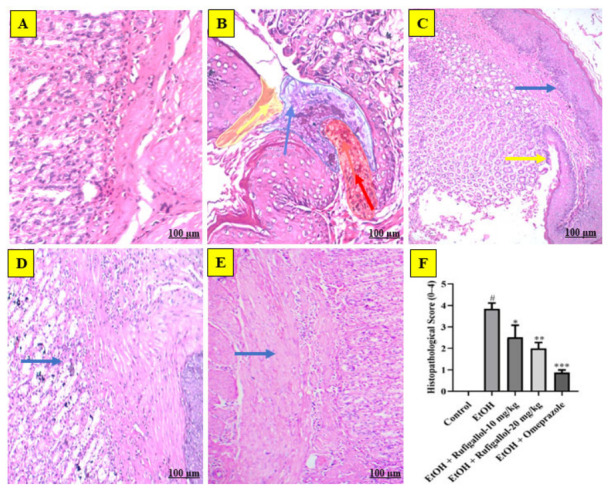
(**A**–**F**). Treatment effect of rufigallol on the appearance of the stomach mucosa in ethanol-induced ulcerogenic rats (magnification: 200×; scale bar = 100 μm). (**A**) Control—Control untreated, (**B**) EtOH: Gastric ulcer induced group only ethanol treated with gastric Lesion (eroded, inflamed region showing epithelial loss, Yellow arrow), mucosal Damage (disrupted mucosal lining and cellular disorganization, Blue arrow), necrosis (dark, fragmented tissue with cellular debris and inflammatory Red arrow), (**C**) EtOH + Rufigallol-10 mg/kg—Ethanol with co-treatment of 10 mg/kg rufigallol (Moderate gastric tissue damage, Blue and Yellow arrow), (**D**) EtOH + Rufigallol-20 mg/kg- Ethanol with co-treatment of 20 mg/kg rufigallol (mild gastric tissue damage and fewer mucosal penetration, Blue arrow), and (**E**) EtOH + Omeprazole—Ethanol with omeprazole (20 mg/kg) treatment (Near to normal gastric tissue damage and fewer mucosal penetration, Blue arrow) (**F**) Histopathological Score-A one-way ANOVA followed by Tukey’s post hoc test, # *p* < 0.001 EtOH vs. control; * *p* < 0.05, ** *p* < 0.01, *** *p* < 0.001 rufigallol/omeprazole treated group vs. EtOH.

Figure 8A–F depict how rufigallol influences ethanol-induced gastric damage in rat models. The histopathological analysis of both the control group reveals that the gastric tissue maintained an intact mucosa, with a well-structured epithelium and properly formed gastric glands. Conversely, rats exposed to EtOH exhibited severe gastric lesions, characterized by significant degeneration, necrosis, hemorrhage, a substantial influx of inflammatory cells, and submucosal edema throughout the gastric wall. Nonetheless, administering rufigallol and the test drug omeprazole notably mitigated these pathological changes induced by EtOH. Specifically, rufigallol treatment decreased the infiltration of inflammatory cells and submucosal edema in a dose-dependent fashion. Remarkably, doses of 10 mg/kg and 20 mg/kg of rufigallol significantly (*p* < 0.01) improved gastric histopathological conditions while maintaining gastric structure. The protective effect of rufigallol was most evident at the highest dose of 20 mg/kg.

### 3.10. MTT Assay and Quantification of Cytokines in the HT-29 Cell Line

**Figure 9:**
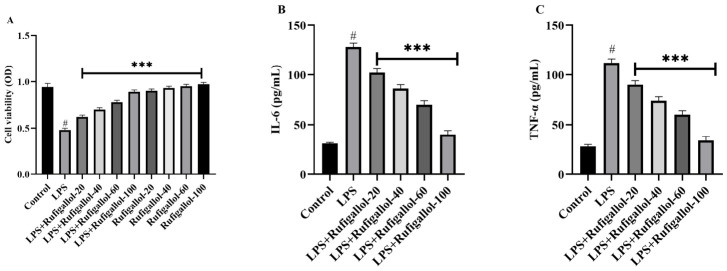
(**A**–**C)**. Effect of rufigallol (20 to 100 μg/mL) treatment on (**A**) Cell viability, (**B)** IL-6 secretion, and (**C**) TNF-α secretion in LPS (10 μg/mL)-stimulated cells. TNF-α, tumor necrosis factor alpha; IL-6, interleukin 6; LPS, lipopolysaccharide. Data are presented as mean ± SEM from independent triplicate experiments. Statistical significance was analyzed by one-way ANOVA followed by Tukey’s post hoc test # *p* < 0.001 LPS vs control; *** *p* < 0.001 rufigallol-treated group vs LPS. Vehicle control (0.2% DMSO), LPS-only control, and rufigallol-alone control were included as appropriate experimental groups.

Figure 9A–C illustrates the impact of rufigallol on cell viability, IL-6 secretion, and TNF-α secretion in LPS-stimulated cells. To identify the most effective concentrations of rufigallol and LPS, HaCaT cells were exposed to varying concentrations of each, and their viability was measured using the in vitro assay. The results show that LPS (10 µg/mL) significantly decreased cell viability (*p* < 0.001). On the other hand, rufigallol at concentrations of 20–100 μg/mL did not adversely affect cell viability compared with the control group. Vehicle-treated cells showed no significant difference in cell viability compared with untreated control cells (*p* > 0.05), indicating that the solvent had no detectable cytotoxic effect. The combination of LPS and rufigallol significantly increased cell viability (*p* < 0.001) and reduced the effects of LPS. Figure 9B,C shows the production levels of TNF-α and IL-6 in the supernatant of HaCaT cells, indicating that the LPS-exposed group had significantly higher TNF-α and IL-6 levels than the control group (*p* < 0.001). On the other hand, the groups treated with both LPA and different concentrations of rufigallol showed a significant reduction in TNF-α and IL-6 levels. This increase is significant, indicating a dose-dependent effect. Notably, cells treated with 100 µg/mL rufigallol restored TNF-α and IL-6 expression levels to levels similar to those of the control group.

## 4. Discussion

Peptic ulcers are one of the most common gastrointestinal disorders in the world, affecting people of all ages and in all parts of the world. Ethanol consumption directly damages the gastric mucosa, leading to oxidative stress, inflammation, and impairment of the protective gastric mucosal barrier. This damage is manifested as hemorrhagic necrosis, submucosal edema, epithelial cell destruction, inflammatory responses, and a reduction in the mucus glycoprotein content of the gastric mucosa [4]. These pathological changes have been consistently documented in animal studies that simulate human gastric ulcers, thereby validating EI-GU as a globally applicable experimental model for evaluating potential gastroprotective agents [29]. Proton-pump inhibitor drugs, like omeprazole, are one of the most commonly used drugs to treat EI-Gus all over the world. However, such drugs have several side effects, as they do not fully heal the ethanol-generated damage, which has led to research into other treatments that are safer and more effective. This has led to the exploration of various natural compounds worldwide that exhibit potent antioxidant, anti-inflammatory, and anti-apoptotic properties and can counter ethanol’s detrimental effects on the gastric mucosa [4,6,8,30]. Anthraquinone derivatives have been extensively studied for their ability to protect the stomach and heal ulcers. Natural derivatives such as emodin, chrysophanol, and physcion demonstrate considerable therapeutic promise in treating experimentally induced gastric ulcers by engaging various anti-inflammatory and antioxidant mechanisms [31,32,33].

In numerous in vivo studies on EI-GU, the research typically spanned over 7 days; thus, a 7-day duration was chosen for this study [22,34]. The present study assessed the application of rufigallol, a compound from the hydroxyanthraquinone family and a derivative of gallic acid, as a test product for the treatment of gastric ulcer [35,36]. The acute oral toxicity study and the safety and pharmacokinetic profile of rufigallol were initially assessed using the pkCSM platform, which predicts ADMET properties. Although these computational predictions offer useful early insights into the drug-likeness and potential safety of rufigallol, they should be viewed as supportive evidence rather than definitive experimental proof [20]. Research on EI-GU in rats and mice consistently indicates disruption of the gastric mucosal barrier, resulting in pathological changes such as an elevated ulcer index, altered gastric pH, and reduced pepsin activity. Specifically, ethanol administration significantly enhanced the gastric ulcer index, reflecting the extent of mucosal damage [6,37,38,39]. Studies have demonstrated that ethanol substantially decreases the gastric pH by enhancing gastric juice acidity and volume, while diminishing mucosal protective factors such as mucus and prostaglandins [37,40]. In the current study, omeprazole and rufigallol were assessed against EI-GUs and substantially lowered the ethanol-induced ulcer index, indicating their protective effects in the gastric mucosa. Treatment also raised the pH and reduced the amounts of gastric juice, free acidity, and total acidity. At the same time, the ulcer index also decreased. Ethanol exposure affects pepsin activity, a key factor in gastric digestion, and could worsen ulcers. Ethanol-induced inflammation and oxidative stress can modify pepsin activity levels and its activity [40]. Rufigallol treatment significantly restored pepsin activity levels decreased by ethanol treatment, indicating a protective and normalizing effect on gastric proteolytic function. Studies of EI-GU models in rats and mice have consistently reported elevated ulcer index scores characterized by macroscopic and microscopic mucosal lesions following ethanol exposure.

Several studies have confirmed that the pathogenesis of ethanol-induced gastric ulcers involves oxidative stress, inflammation, neutrophil infiltration, reduced mucus secretion, apoptosis in the gastric mucosa, and disruption of the mucosal barrier. Chronic alcohol consumption perpetuates redox imbalance across the gut–liver axis. This prolonged intestinal ethanol uptake provokes oxidative stress by inducing cytochrome P450 activity in the intestinal mucosa, a major source of ROS, leading to lipid peroxidation (increased MDA levels) and depletion of antioxidants (GSH) and associated enzymes (CAT and SOD). Mitochondrial oxidative stress triggers intrinsic apoptotic pathways by modulating pro- and anti-apoptotic proteins and releasing cytochrome c and apoptosis-inducing factor, which subsequently disrupts epithelial barrier integrity, leading to increased permeability (leaky gut) [4,29,41]. Anthraquinone derivatives show antioxidant activity primarily by scavenging free radicals that drive oxidative stress. Six related anthraquinones, alizarin, purpurin, chrysophanol, emodin, aloe-emodin, and 1,3,8-trihydroxyanthraquinone, have been reported for radical-inactivating efficiency [42]. Their antioxidant activity helps restore redox homeostasis by directly scavenging radicals involved in damaging processes and is crucial in many neurodegenerative, cardiovascular, and inflammatory diseases [43]. Anthraquinone derivatives may also enhance antioxidant defenses by modulating related cellular systems and activating SOD and CAT through distinct pathways, thereby increasing endogenous protection against oxidative injury [44]. Our research findings also show similar results: elevated MDA levels in the ethanol-treated group, along with remarkable reductions in SOD, GSH, and CAT levels, highlighting ethanol’s role in lipid peroxidation. Additionally, treatment with rufigallol (10 and 20 mg/kg) and omeprazole significantly reduces MDA activity while restoring GSH, SOD, and CAT levels in the experimental groups.

PGE_2_ in EI-GU is centered on its role as a critical mediator of mucosal defence. PGE_2_ supports antioxidant defenses, modulates inflammatory responses, enhances mucous secretion, and improves microcirculation, all of which contribute to protection against ethanol-induced damage and ulceration [11,12]. Treatment with rufigallol also helps restore or stimulate PGE_2_ levels, which contributes to blocking or reducing mucosal injury, oxidative stress, and inflammation in experimental models of ethanol-induced gastric ulcers.

Studies have confirmed that oxidative stress and IFN-γ play notable roles in the pathogenesis of ethanol-induced gastric ulcers by promoting the production of proinflammatory cytokines, such as TNF-α, IL-6, and IL-1β, via macrophage activation, thereby exacerbating mucosal damage. Several natural and pharmacological agents counteract these changes by suppressing inflammatory cytokines (TNF-α, IL-1β) and NF-κB activation [6,37,39,40,45]. The present research, building on this finding, assesses this proinflammatory cytokine in ethanol-induced rat models and investigates the effects of rufigallol on it. Administration of rufigallol in rats significantly improved ethanol/HCl-induced gastric mucosal injury, and this result was prominently linked to the downregulation of NF-κB signaling and reduced mRNA expression of inflammation-related genes, including IFN-γ, TNF-α, and NF-κB [9]. In vitro research determined the optimal concentrations of LPS and rufigallol for HaCaT cells, revealing that the various rufigallol concentrations tested did not exhibit significant cytotoxicity. Conversely, rufigallol treatment mitigated LPS-induced effects in HaCaT cells and enhanced cell survival. In the in vitro HaCaT cell model stimulated by LPS, there was an increase in TNF-α and IL-6 levels. Rufigallol notably reduced the secretion of the inflammatory cytokines TNF-α and IL-6 in vitro. These findings indicated that rufigallol could alleviate inflammation by suppressing the secretion of TNF-α and IL-6. Omeprazole therapy also decreases inflammatory mediators such as TNF-α, IL-6, and IL-1β, including NLRP3, by downregulating NF-κB signaling pathways, thereby reducing gastric mucosal injury and inflammation following ethanol challenge [30]. Treatment with rufigallol at both 10 mg/kg and 20 mg/kg was significant across all previously mentioned parameters. Studies specifically addressing anthraquinone derivatives show that they prevent ethanol-induced gastric ulcers mainly through strong gastroprotective and anti-inflammatory effects, rather than by stimulating inflammation. They safeguard the gastric mucosa by actively reducing the production of cytokines and inhibiting the excessive activation of the inflammatory cells. They exert anti-ulcer activity largely by preventing the depletion of gastric mucosal defensive factors, notably PGE_2_ and COX-1, while simultaneously downregulating pro-inflammatory markers [46]. As seen in EI-GUs, the defensive effects induced by the application of natural pharmaceutical treatments are associated with upregulation of endothelial nitric oxide synthase, which restores NO levels and downregulates MPO-mediated neutrophil recruitment in gastric mucosal tissue, helping to maintain mucosal blood flow and reduce neutrophil infiltration, thus ultimately reducing inflammation [4,39,47]. The results of the current study suggest that ethanol administration leads to an increase in MPO activity and a decrease in NO activity. These effects are mitigated by treatment with rufigallol in experimental rat models. Similar to these results, other anthraquinones provide significant gastroprotection against gastric ulcers by suppressing MPO activity and modulating NO pathways [48].

EI-GU involves complex inflammatory and oxidative mechanisms, with NF-κB and COX-2 playing crucial roles in its pathogenesis. At a molecular level, acute ethanol exposure leads to ROS generation in animal models, activating NF-κB signaling and promoting the expression of proinflammatory mediators, including COX-2, in gastric tissues, thereby activating the inflammatory cascade. The interplay between oxidative stress and NF-κB/COX-2 activation creates a vicious cycle that disrupts gastric mucosal integrity, promotes ulceration, and induces apoptosis [49,50,51]. Treatment with rufigallol, an antioxidant, concurrently inhibits NF-κB activation and COX-2 expression in gastric cells by restoring the balance between oxidative stress and decreased lipid peroxidation, fewer apoptotic cells, and restoration of gastric mucosal integrity and mucus production.

Omeprazole served as the reference anti-ulcer agent because of its well-established gastroprotective efficacy [30]. Compared with the ethanol-treated group, both rufigallol and omeprazole significantly reduced gastric injury, ameliorated histopathologic changes, attenuated oxidative stress, and suppressed inflammatory mediators. The gastroprotective effects of rufigallol were dose-dependent, with the 20 mg/kg dose consistently providing greater protection than the 10 mg/kg dose. Although omeprazole generally showed the strongest response across the parameters evaluated, the higher dose of rufigallol showed comparable improvement in several outcomes, suggesting substantial pharmacological activity. These findings suggest that rufigallol has promising gastroprotective properties, while recognizing that direct equivalence to omeprazole cannot be established without dedicated comparative pharmacological and dose–response studies.

**Figure 10:**
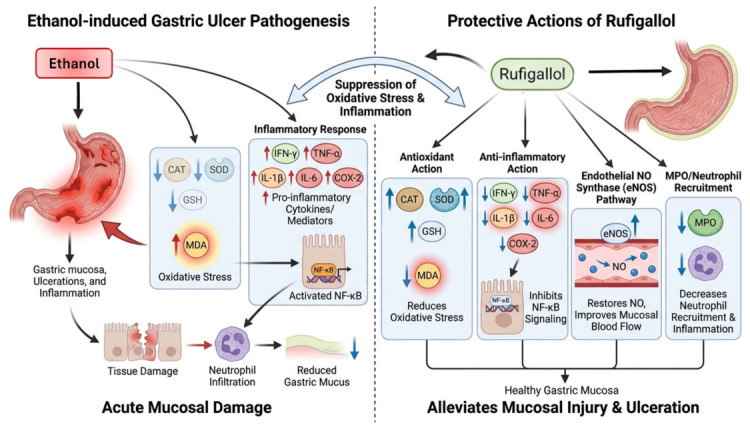
Proposed hypothetical mechanism of rufigallol action against the ethanol-induced gastric ulcer.

Based on these results, we propose a possible mechanism of action for rufigallol treatment. Ethanol induces gastric ulcers by promoting oxidative stress and triggering inflammation, characterized by decreased levels of different oxidative stress markers (CAT, SOD, and GSH) and increased lipid peroxidation process (by MDA level) and production of pro-inflammatory cytokines such as IFN-γ, TNF-α, IL-1β, and IL-6, and mediators like COX-2. This process causes tissue damage, neutrophil infiltration, and reduced gastric mucus, resulting in acute mucosal injury. Rufigallol often acts by suppressing both oxidative stress and inflammatory pathways, including downregulation of NF-κB and NLRP3 signaling, and may reduce the levels of inflammation-related molecules, including IFN-γ, TNF-α, NF-κB, and NLRP3, thereby significantly alleviating ethanol-induced gastric mucosal injury in rats. It is also proposed that the involvement of IFN-γ in EI-GUs is closely linked to its role as a pro-inflammatory cytokine that exacerbates local inflammation and epithelial damage via NF-κB-dependent pathways. Rufigallol may also upregulate endothelial nitric oxide synthase, thereby restoring NO levels and downregulating MPO-mediated neutrophil recruitment in gastric mucosal tissue, helping to maintain mucosal blood flow and reduce neutrophil infiltration, ultimately reducing inflammation (Figure 10). To our knowledge, this is the first study to comprehensively demonstrate the gastroprotective potential of rufigallol against ethanol-induced gastric injury by integrating macroscopic, biochemical, inflammatory and histopathological assessments in vivo, together with complementary in vitro evidence of cytokine suppression. Unlike previously investigated anthraquinones, rufigallol demonstrated protective effects across oxidative stress, inflammatory signaling and tissue injury endpoints, and its efficacy was evaluated in comparison to the standard drug omeprazole, supporting its promise as a novel gastroprotective candidate.

The present findings suggest that the gastroprotective effects of rufigallol involve modulation of COX-2, IFN-γ, TNF-α, IL-1β, IL-6, PGE_2_, NF-κB, and NLRP3-associated inflammatory responses. However, since ELISA was used to evaluate these markers, the data reflect an association with these pathways rather than direct mechanistic regulation. The absence of Western blotting, immunohistochemistry, and PCR analysis limits the depth of mechanistic interpretation. These techniques were not included because the primary goal of this pilot study was to generate preliminary data and inform the design of larger-scale future investigations. Such techniques require specialized facilities, biological samples, and ethical approval for animal use, all of which were beyond the scope of the current study.

Although rufigallol appears to exert gastroprotective effects by modulating oxidative stress and inflammatory pathways, this study did not directly validate the upstream molecular mechanisms. The in vitro experiments focused on anti-inflammatory activity through cytokine measurements. Oxidative stress markers and antioxidant signaling were not assessed at the cellular level. While the suppression of IL-6 and TNF-α production confirmed an anti-inflammatory effect in vitro, other mediators examined in the in vivo component, including IL-1β, NF-κB, and NLRP3, were not investigated at the cellular level. Therefore, the in vitro findings should be regarded as supportive evidence rather than mechanistic proof. The in vitro experiments used a non-gastric cell model that, while well-established for studying LPS-induced inflammation, does not fully replicate gastric epithelial cell characteristics. Future work should validate these findings using gastric epithelial cell lines, such as AGS or GES-1, and include experiments that clarify the direct role of rucinol in mucosal protection.

Future studies should employ strategies that target specific pathways, including inhibitors and agonists, as well as gene expression analyses and cellular oxidative stress assays. These assays should include intracellular ROS measurements and evaluation of Nrf2/HO-1 signaling to more fully elucidate the mechanism of action of rufigallol.

The gastric mucus barrier is a key component of mucosal defense against ethanol-induced injury; however, this study did not directly assess mucus production or thickness. Future studies should incorporate evaluations specific to mucus and specialized histological staining, such as PAS/Alcian blue for mucus and Masson’s trichrome for collagen deposition, to better characterize rufigallol’s protective effects on the gastric mucosa. Additionally, although the ethanol-induced gastric ulcer model is commonly used to evaluate gastroprotective agents and acute mucosal injury, it does not capture the full complexity or chronic progression of human gastric ulcer disease. Therefore, the translational relevance of these findings should be interpreted with appropriate caution. The observed improvements in gastric injury, oxidative stress, inflammatory mediators, and histopathological parameters provide preliminary evidence warranting further mechanistic, long-term preclinical, and translational investigations before considering any clinical applications.

## 5. Conclusions

In conclusion, EI-GUs are a significant global health concern intricately associated with alcohol consumption patterns worldwide. Rufigallol, an anthraquinone derivative, protected against ethanol-induced gastric injury in rats. This was evidenced by a lower ulcer index, improved gastric pH, reduced gastric volume and pepsin activity, and attenuated histopathological damage. Rufigallol also reduced lipid peroxidation, restored antioxidant defenses, and decreased inflammatory cytokines and mediators in gastric tissue. In vitro, rucinol reduced IL-6 and TNF-α production in stimulated cell-based assays. Overall, these data support the use of rufigallol as a promising gastroprotective agent, though further studies are needed to confirm its broader translational potential.

## Figures and Tables

**Table 1:** Detailed marking of the ulcer index based on the evaluation index.

Evaluation Index	Scores
Without lesions (normal stomach)	0
Hyperaemia	0.5–1
Haemorrhagic spots	1–2
1–5 small ulcers	2–3
Several small ulcers	3–4
1–5 small and 1–3 large ulcers	4–5
Several small and large ulcers	5–6
Stomach full of ulcers or perforations	6

**Table 2:** Effect of rufigallol on gastroprotection in ethanol-induced rats.

Group	Protection Index (%)
Control	100.00
EtOH	0.00
EtOH + Rufigallol (10 mg/kg)	69.98
EtOH + Rufigallol (20 mg/kg)	77.58
EtOH + Omeprazole	81.11

## Data Availability

No new data were created or analyzed in this study. Data sharing is not applicable to this article.
